# Research progress and future perspectives in proteolysis-targeting chimeras

**DOI:** 10.3389/fphar.2026.1815569

**Published:** 2026-05-20

**Authors:** Nuofan Wu, Zihui Xu, Shuhong Dai, Qianqian Wang

**Affiliations:** 1 School of Food and Drug, Shenzhen Polytechnic University, Shenzhen, China; 2 School of Biological Science and Engineering, South China University of Technology, Guangzhou, China

**Keywords:** clinical translation, E3 ubiquitin ligase, pharmacokinetics, proteolysis-targeting chimera (PROTAC), targeted protein degradation (TPD), ubiquitin-proteasome system

## Abstract

Proteolysis-targeting chimeras (PROTACs) have emerged as a transformative modality within the targeted protein degradation (TPD) landscape, inducing spatial proximity between E3 ubiquitin ligases and proteins of interest (POIs) to hijack the ubiquitin-proteasome system (UPS). Unlike traditional occupancy-driven inhibitors, this catalytic, event-driven mechanism enables the targeting of historically “undruggable” proteomes and circumvents acquired resistance. However, the field faces formidable challenges, including suboptimal pharmacokinetic profiles, the stoichiometric “hook effect,” and a disproportionate reliance on a limited pool of E3 ligases (notably cereblon (CRBN) and von Hippel-Lindau (VHL)). This review critically examines PROTAC core principles and provides a nuanced functional categorization across oncology, immune modulation, neurodegenerative diseases, and basic research. We further evaluate three pivotal technical strategies—degrader architecture innovations, conditional activation modalities, and advanced delivery platforms—while systematically appraising current clinical progress. Finally, we discuss key limitations and future translational directions, aiming to provide a realistic roadmap for the next-generation of TPD therapeutics.

## 1 Introduction

The escalating global burden of cancer remains a critical public health challenge, with approximately 20 million newcases and 9.7 million deaths reported in 2022. The disproportionate rise in mortality within low- and middle-income regions (Bray et al., 2024) highlights a widening gap in therapeutic accessibility and efficacy, underscoring the need for more effective and feasible strategies. While traditional chemotherapy provides systemic cytostatic effects, its inherent lack of selectivity often results in dose-limiting toxicities that compromise patient quality of life (Manavi et al., 2024). The shift toward precision-targeted therapies, such as small-molecule inhibitors (SMIs), substantially advanced oncology by employing an occupancy-driven mechanism (Hanahan and Weinberg, 2011). However, the therapeutic ceiling of this paradigm is increasingly evident.

This occupancy-driven paradigm suffers from fundamental pharmacological constraints. First, achieving therapeutic efficacy often requires sustained near-complete saturation of the target’s active site, which frequently necessitates high systemic exposure and heightens the risk of off-target effects (Bond and Crews, 2021). Second, SMIs are largely restricted to proteins with well-defined catalytic or hydrophobic pockets, leaving recalcitrant targets—such as transcription factors and non-enzymatic scaffolds—effectively “undruggable” (Chirnomas et al., 2023). Third, and most crucially, the selective pressure exerted by chronic inhibition inevitably triggers acquired resistance (Ma et al., 2024). This is exemplified by the EGFR T790M mutation in lung cancer, which restores ATP-binding affinity and renders gefitinib ineffective (Pao et al., 2005; Housman et al., 2014). Unlike simple competitive inhibition, resistance in the SMI era is often a sophisticated adaptive response involving target genomic remodeling or bypass signaling activation.

These limitations have catalyzed the transition from protein inhibition to TPD. Unlike SMIs, TPD leverages cellular machinery—primarily the lysosomal or UPS—to physically eliminate disease-causing proteins. PROTACs represent the most mature UPS-based platform, utilizing an event-driven catalytic cycle to induce POI ubiquitination. By decoupling the requirement for high-affinity active-site binding from therapeutic outcome, PROTACs enable the degradation of proteins previously dismissed as undruggable. Notably, while the high molecular weight of PROTACs initially raised concerns regarding “Beyond Rule of 5”(bRo5) pharmacokinetic profiles, recent engineering has yielded orally bioavailable candidates such as Vepdegestrant (ARV-471) and KT-474, which have entered late-stage clinical trials (Rallabandi et al., 2025; Pike et al., 2026). Nevertheless, the clinical transition of PROTACs is not without hurdles; issues such as stoichiometric imbalance and complex PK/PD relationships remain central to ongoing discourse.

This review critically evaluates the molecular mechanisms and structural determinants of PROTAC technology. We provide a functional categorization based on functional applications across oncology, immunology, and neurology. Furthermore, we analyze current technical strategies, including degrader architecture diversification, conditional activation modalities, and delivery-enhanced platforms. By synthesizing current clinical data and addressing inherent limitations such as resistance and bioavailability, this work aims to provide a comprehensive yet realistic roadmap for the future of targeted protein degradation.

## 2 Core principles and overview of PROTAC technology

### 2.1 Core definition

**FIGURE 1:**
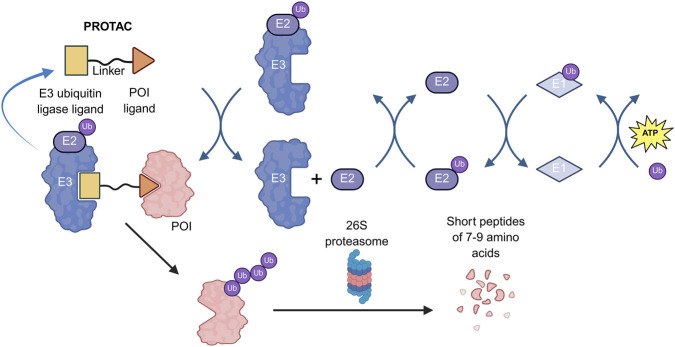
Schematic diagram of the mechanism of PROTACs. This diagram illustrates the core event-driven mechanism of PROTACS, depicting the process by which PROTAC molecules bridge target proteins and E3 ubiquitin ligases to form a ternary complex, thereby inducing ubiquitination and subsequent proteasomal degradation of pathogenic target proteins.

PROTACs are heterobifunctional molecules that harness the intracellular UPS to degrade proteins of interest. A typical PROTAC comprises three elements: a ligand binding the POI, a ligand recruiting an E3 ubiquitin ligase, and a chemical linker. As shown in Figure 1, the PROTAC acts as a “molecular bridge,” inducing spatial proximity between the POI and the E3 ligase to form a ternary complex, which subsequently triggers POI polyubiquitination and degradation by the 26S proteasome (Békés et al., 2022).

To fully appreciate this mechanism, it is instructive to contrast PROTACs with molecular glues—another major class of UPS-harnessing degraders. While both operate catalytically, molecular glues (e.g., thalidomide derivatives) are monovalent small molecules that reshape the E3 ligase surface to induce a novel protein-protein interaction (PPI) with the target, requiring no linker (Oleinikovas et al., 2024; Metwally et al., 2025). Consequently, molecular glues are structurally simpler but traditionally discovered serendipitously with a restricted target scope. In contrast, PROTACs are rationally designable and modular, affording them broad applicability across the TPD landscape for historically “undruggable” targets (Eladl, 2025; Khaledian et al., 2025).

### 2.2 Development timeline and mechanism of action

**FIGURE 2:**
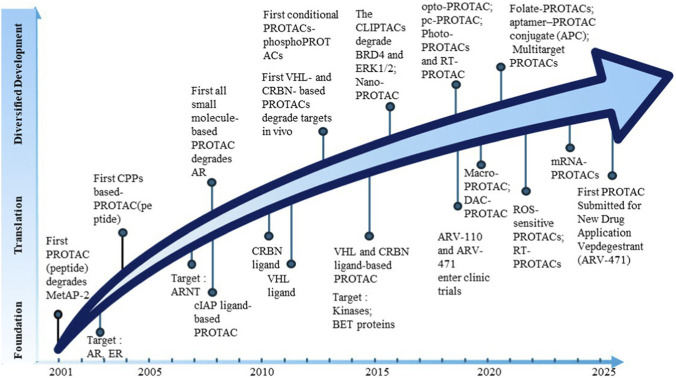
Developmental history of PROTACs. This figure outlines the key evolutionary stages of PROTAC technology from its conceptual proposal in 2001 to the current clinical translation phase, highlighting critical milestones such as the transition from peptide-based prototypes to fully small-molecule PROTACs, the validation of core mechanisms, and the advancement of representative candidates into clinical trials.

Since the proof-of-concept peptide PROTAC was reported in 2001 (Sakamoto et al., 2001), the field has rapidly transitioned to fully small-molecule therapeutics, driven by the discovery of potent CRBN and VHL ligands and culminating in the clinical advancement of candidates like ARV-110 and ARV-471 (Smith et al., 2008; Ito et al., 2010; Ma et al., 2024). At the heart of this clinical translation is the PROTAC-mediated degradation mechanism—a dynamic, catalytic cycle exploiting the native UPS. Figure 2 illustrates the development of PROTAC technology very well.

As illustrated in Figure 1, the cycle initiates when a PROTAC engages both the POI and an E3 ligase to assemble a POI-PROTAC-E3 ternary complex. Facilitated by spatial proximity, a ubiquitin cascade involving E1 and E2 enzymes transfers ubiquitin molecules to surface-exposed lysine residues on the POI. Following successful polyubiquitination, the ternary-complex dissociates, releasing the intact PROTAC to participate in subsequent catalytic rounds. The ubiquitinated POI is then recognized by the 26S proteasome, unfolded by ATP-dependent unfoldases, and translocated into the 20S core particle for proteolytic cleavage (Ma et al., 2024).

### 2.3 Key features

The event-driven nature of PROTACs yields distinctive pharmacological attributes, most notably their catalytic efficiency. A single PROTAC molecule can mediate the sequential degradation of multiple POIs, achieving potent target suppression at sub-stoichiometric concentrations and minimizing off-target toxicity (Bondeson et al., 2015; Békés et al., 2022). However, direct *in vivo* evidence for this catalytic recycling remains limited.

However, this bivalent nature inherently introduces the “hook effect”—a stoichiometric trap where excess PROTAC molecules saturate both POI and E3 ligases independently, forming non-productive binary complexes that paradoxically attenuate degradation efficiency at high doses (Islam et al., 2025). Overcoming this limitation requires rational design to favor ternary over binary complex formation. Macrocyclization, for instance, introduces conformational constraints that thermodynamically drive ternary assembly (Li et al., 2024).

Beyond concentration dynamics, degradation efficacy fundamentally relies on the thermodynamic stability and cooperativity of the ternary complex. Cooperativity dictates whether the PROTAC-induced POI-E3 engagement is mutually reinforcing. Recent structural insights reveal that energetic frustration at the neo-PPI interface positively regulates this cooperativity, providing a critical parameter for degrader optimization (Ma et al., 2025). Furthermore, geometric prediction tools like Deep Ternary confirm that maximizing the buried surface area correlates robustly with degradation potency (Xue et al., 2025).

In this context, the chemical linker transcends its role as a mere physical tether; it acts as a spatial gatekeeper. By dictating the relative orientation and inter-protein distance, linker length and rigidity directly govern the entropic cost of complex formation (Cao et al., 2026). For instance, rigid triazole-containing linkers have been shown to stabilize ternary complexes and enhance cooperativity, underscoring linker optimization as a decisive determinant in PROTAC success (Shehzadi et al., 2025).

Despite these optimizations, the durability of PROTAC therapy is continually challenged by the evolutionary pressure of acquired resistance. The disproportionate reliance on single E3 ligases (e.g., CRBN) frequently selects for loss-of-function genomic alterations in the ligase machinery. Additionally, while PROTACs like NX-2127 effectively overcome classical inhibitor-resistant mutations (e.g., BTK C481S), they impose selective pressures that breed novel POI mutations (e.g., BTK L528W) which disrupt degrader binding or function (Huynh et al., 2024; Sabakhtarishvili et al., 2025). Mitigating this resistance demands next-generation strategies, such as multivalent degraders capable of recruiting dual E3 ligases, thereby maintaining target ubiquitination even if one pathway is compromised (Spiteri et al., 2025).

## 3 Functional classification of PROTAC technology

**FIGURE 3:**
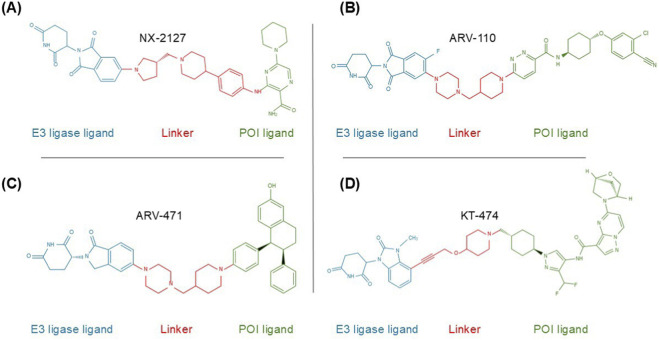
Chemical structures of representative PROTACs. The figures display the three-core modular architecture (E3 ligase ligand, linker, and POI ligand) of four leading PROTAC candidates: **(A)** NX-2127 targets BTK with a pyrrolidine-based linker; **(B)** ARV-110 targets AR featuring a piperazine-based linker; **(C)** ARV-471 targets ER with a chiral cyclohexane linker; **(D)** KT-474 targets IRAK4 utilizing a triazole-containing linker. The color coding highlights the E3 ligase ligand (blue), linker (red), and POI ligand (green).

The broad therapeutic potential of PROTACs stems from their capacity to address fundamental limitations of occupancy-driven pharmacology. In this chapter, we pivot from structural chemistry to functional application scenarios. By classifying PROTACs according to their disease-specific implementations—spanning oncology, immune modulation, neurology, and synthetic biology—we critically examine how this modality reshapes the therapeutic landscape, while highlighting the distinct biological and pharmacokinetic hurdles inherent to each domain. Meanwhile, we will present the chemical structures of several representative PROTACs to enhance the readers’ understanding of PROTACs (see Figure 3 for details).

### 3.1 PROTACs in oncology

Oncology remains the most clinically advanced arena for PROTAC technology, spearheaded by candidates like the FDA-submitted ARV-471 (Vepdegestrant) and various Bruton’s tyrosine kinase (BTK)/Androgen receptor (AR) degraders entering late-stage trials (Ma and Zhou, 2025; Vikal et al., 2025). While much of this progress has been driven by well-characterized nuclear receptors and kinases—benefiting from the historical availability of high-affinity ligands—the field is actively expanding its scope.

Notably, numerous entities are pursuing preclinical investigations targeting other conventionally “undruggable” proteins, including KRAS mutants and PI3K, demonstrating that PROTAC technology is progressively moving beyond its initial comfort zone (Tsai et al., 2024). Nevertheless, short-lived cytosolic proteins, transient metabolic enzymes, and dynamically disordered scaffolding proteins remain comparatively underexplored (Samarasinghe et al., 2021; Békés et al., 2022). Furthermore, the clinical pipeline exhibits a fragile monopolistic reliance on CRBN as the recruited E3 ligase. Emerging clinical data reveal that this over-reliance exerts a selective pressure, driving acquired resistance through CRBN genomic mutations or epigenetic downregulation (Hanzl et al., 2023). To overcome these bottlenecks, the field should pivot towards expanding the E3 ligase repertoire (e.g., tumor-specific E3 recruitment) and optimizing linker kinetics to tackle rapid-turnover proteomes.

### 3.2 PROTACs in immune modulation and inflammatory diseases

Beyond oncology, PROTACs are advancing the management of immune and inflammatory responses. In cancer immunotherapy, degradation of immune checkpoints (e.g., Programmed Death-Ligand 1 (PD-L1)/Programmed Death-1 (PD-1)) not only abrogates receptor-ligand interactions but eradicates the protein entirely, circumventing the compensatory upregulation pathways that frequently plague monoclonal antibody therapies (Li et al., 2022; Moon et al., 2025). Moreover, precise spatiotemporal immune regulation has been achieved using photoactivatable PROTACs to remotely modulate Chimeric Antigen Receptor T (CAR-T) cell activity, ensuring on-demand activation of engineered therapeutic cells (Sharma et al., 2025).

In the autoimmune sector, KT-474 has successfully translated to Phase II trials for hidradenitis suppurativa and atopic dermatitis by degrading Interleukin-1 Receptor-Associated Kinase 4 (IRAK4) in the IL-1R/TLR pathway (Zheng et al., 2024). However, the unique pharmacological profile of PROTACs presents a double-edged sword in immunology. Because degradation triggers a prolonged pharmacodynamic (PD) effect that severely outlasts the drug’s pharmacokinetic (PK) clearance, PROTACs cannot be rapidly reversed by simple drug washout (Samarasinghe and Crews, 2021). In the context of acute immune-related adverse events (irAEs) or cytokine storms, this irreversible immunosuppression poses a severe clinical risk. Addressing this necessitates the integration of conditionally controllable degraders and tissue-restricted delivery platforms to strictly confine degradation to pathological niches.

### 3.3 PROTACs in neurodegenerative diseases

Neurodegenerative diseases represent a highly anticipated, yet profoundly challenging, frontier for TPD. While ARV-102 (Leucine-Rich Repeat Kinase 2 (LRRK2) degrader) has completed Phase I trials and preclinical Tau-targeted PROTACs have demonstrated cognitive rescue in Alzheimer’s models (Yao et al., 2024), bridging the gap between targeted degradation and meaningful neuromodulation remains an arduous task.

The central bottleneck is a physicochemical paradox: PROTACs inherently possess high molecular weights (>600 Da) and topological polar surface areas (TPSA) that severely restrict passive diffusion across the blood-brain barrier (BBB). While some preclinical studies artificially bypass this via intracranial injection, such methods lack broad clinical translatability (Wang C. et al., 2023; Kong et al., 2025; Francisco et al., 2026). Even upon successful CNS entry, a secondary biological barrier emerges: proteotoxicity. Pathological proteins in neurodegeneration (e.g., misfolded Tau, α-synuclein) often assemble into dense oligomers or amyloid aggregates. These quaternary structures not only mask potential ubiquitination sites via steric hindrance but are also intrinsically resistant to the proteolytic unwinding machinery of the 26S proteasome. Consequently, future neuro-PROTACs must be rationally engineered not merely to penetrate the brain, but to selectively recognize disease-specific pathogenic conformations before irreversible aggregation occurs.

### 3.4 PROTACs in basic research and synthetic biology

As highly precise “chemical genetics” tools, PROTACs offer distinct advantages over traditional genetic ablation (e.g., CRISPR-Cas9). They provide rapid, post-translational protein knockdown and remarkable reversibility, preserving cell viability in hard-to-transfect primary cultures (Kanbar et al., 2024). Within synthetic biology, PROTACs have been integrated into sophisticated gene expression circuits. Utilizing AND-gate logic, multi-signal responsive degraders require simultaneous activation by distinct physiological inputs, enabling complex cellular decision-making and logic-gated synthetic responses (Ma et al., 2023; Yang et al., 2025).

Nevertheless, their utility as universal research tools is constrained by the ligandability bottleneck. The design of a PROTAC is strictly predicated on the existence of a high-affinity small-molecule binder for the target protein—a prerequisite lacking for the vast majority of the human proteome. To bypass this, researchers are increasingly adopting ligand-free methodologies, such as nanobody-based bioPROTACs and CRISPR-guided TRAFTACs, alongside high-throughput screening of diverse spatial libraries to democratize access to undruggable targets (Samarasinghe et al., 2021; Zhao S. et al., 2025).

### 3.5 Other emerging disease applications

The PROTAC paradigm is also being explored in nascent clinical territories. Exploratory degraders targeting Proprotein Convertase Subtilisin/Kexin type 9 (PCSK9) for hypercholesterolemia (Fan et al., 2026), glucose regulatory nodes for diabetes (Mobeen et al., 2024), and pro-fibrotic Suppressor of Mothers Against Decapentaplegic (SMAD) complexes for tissue fibrosis (Yang J. et al., 2022) have demonstrated preclinical efficacy. Although trailing behind oncology and immunology in clinical maturity, these applications underscore the broad potential of PROTAC technology.

Realizing this therapeutic vision, however, inherently depends on overcoming the structural and delivery limitations discussed above. The molecular engineering required to enable these functional applications—specifically degrader architecture innovations, conditional activation, and targeted delivery platforms—will be examined in Chapter 4.

## 4 Technical strategies for PROTAC development

The translation of PROTACs from concept to therapeutics depends on rational design, systematic optimization, and targeted delivery. In this chapter, we shift focus from functional outcomes to technical implementation, covering three major strategies: degrader architecture innovations, conditional activation strategies, and delivery-enhanced platforms.

### 4.1 Degrader architecture innovations

**FIGURE 4:**
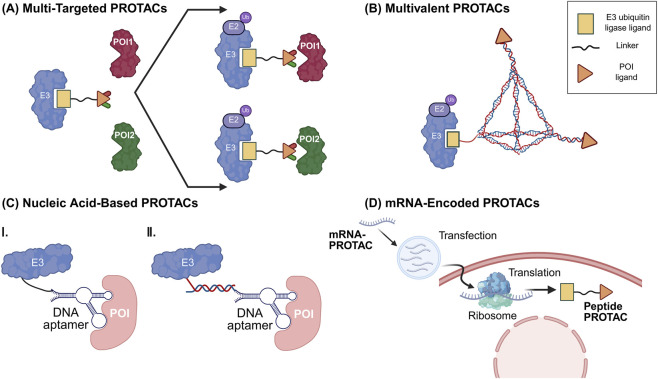
Schematic illustration of design principles and mechanisms of various degrader architecture innovations. **(A)** Multi-targeted PROTACs: A single molecule degrades two or more proteins of interest (POIs) simultaneously to achieve synergistic effects; **(B)** Multivalent PROTACs: Multiple POI ligands are conjugated onto a single scaffold (e.g., DNA tetrahedron) to enhance degradation efficiency via avidity effects, particularly for oligomeric targets or weak-affinity ligands; **(C)** Nucleic acid-based PROTACs: Oligonucleotides (e.g., aptamers); recognize targets lacking conventional binding pockets, such as RNA-binding proteins. I. The E3 ligase and nucleic acid aptamer are conjugated by click reaction; II. The two components are linked by a pair of complementary DNA strands; **(D)** mRNA-encoded PROTACs: Delivery of mRNA encoding a PROTAC enables *in situ* translation and synthesis of the degrader, bypassing permeability limitations of conventional small-molecule PROTACs.

Modern PROTAC design is transcending the classical “one-target, one-ligand” heterobifunctional framework. By engineering higher-order architectures, researchers are now aiming to achieve synergistic multi-target degradation, enhanced binding avidity, and access to the “ligandability” frontier of the human proteome Figure 4 presents the mechanisms of action of some modern PROTACs.

#### 4.1.1 Multi-targeted PROTACs

Multi-targeted PROTACs represent an evolution toward combinatorial therapeutic intelligence. Unlike simple drug cocktails, these single-molecule entities (e.g., dual ERα/aromatase degraders) can achieve synchronized downregulation of interconnected signaling nodes, thereby preventing the compensatory pathway rewiring that frequently drives oncology resistance (Xin et al., 2024; Zhang et al., 2025). Modular platforms, such as the “Multi-Split-and-Mix” system, further facilitate this approach by allowing the rapid assembly of trivalent degraders without the traditional, laborious linker optimization (Wang et al., 2025).

#### 4.1.2 Multivalent PROTACs

For targets with weak ligand affinity or oligomeric quaternary structures, monovalent recruitment often fails to meet the thermodynamic threshold for degradation. Multivalent PROTACs—frequently scaffolded on DNA tetrahedrons—leverage avidity effects to achieve potent degradation where individual ligands would be insufficient (Li et al., 2025). However, the increased molecular size of these constructs imposes a significant “ bRo5” pharmacokinetic penalty, necessitating advanced delivery solutions.

#### 4.1.3 Multi-E3 ligase recruitment

To circumvent “E3-centric” resistance, multifunctional degraders capable of recruiting dual ligases (e.g., CRBN and VHL) act as a biological fail-safe. This redundancy ensures sustained POI ubiquitination even in heterogeneous tumor microenvironments where specific ligase expression may be epigenetically silenced or mutated (Bond et al., 2024; Wang et al., 2025).

#### 4.1.4 Peptide-based PROTACs

Peptide-based PROTACs excel at disrupting large, flat PPI interfaces. A landmark application is the degradation of Breast Cancer Susceptibility Gene 2 (BRCA2), which artificially induces a “BRCAness” phenotype in homologous recombination-proficient cells, thereby creating a *de novo* synthetic lethality vulnerable to PARP inhibitors (Ye et al., 2025). Nevertheless, poor cellular uptake and *in vivo* proteolytic instability remain major barriers to clinical translation.

#### 4.1.5 Nucleic acid-based PROTACs

Leveraging the programmability of oligonucleotides, TRAFTACs and Aptamer-PROTACs engage transcription factors and RNA-binding proteins that lack traditional hydrophobic pockets (Samarasinghe and Crews, 2021). While their modularity is high, their systemic utility is currently tethered by nuclease susceptibility, requiring chemical shielding or nanoparticle encapsulation (Hou et al., 2025).

#### 4.1.6 mRNA-encoded PROTACs

mRNA-PROTACs (e.g., RiboPROTACs) represent a pharmacokinetic paradigm shift. By delivering the “blueprint” (mRNA) rather than the “product” (protein/molecule), this strategy utilizes the cell as a bioreactor to synthesize degraders *in situ*. This bypasses the permeability hurdles of high-molecular-weight PROTACs and offers a promising route for transient, dose-controlled TPD (Yang J. L. et al., 2022; Xue et al., 2024). However, concerns regarding immunogenicity, uncontrolled expression kinetics, and the inability to rapidly terminate degradation remain unresolved.

### 4.2 Conditionally activatable PROTACs

**FIGURE 5:**
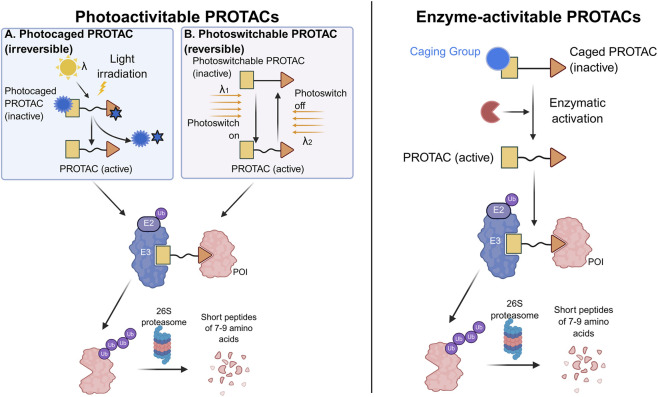
Mechanism of Conditionally Activatable PROTACs. (Left) **(A)** Irreversible photocaged PROTACs release active PROTACs upon irradiation at a specific wavelength, with the photolabile caging group removed; **(B)** Reversible photoswitchable PROTACs realize reversible activity switching through conformational regulation by different wavelengths of light (λ_1_/λ_2_) (Right) Enzyme-activatable PROTACs are activated by specific enzymes in the lesion microenvironment, which cleave the caging group. Activated PROTACs simultaneously bind E3 ubiquitin ligase and the protein of interest (POI), mediate POI ubiquitination, and ultimately degrade POI into short peptides via the 26S proteasome.

To mitigate on-target/off-tumor toxicities, conditionally activatable PROTACs introduce a spatiotemporal “gate” to degradation activity. Figure 5 shows several representative PROTACs that utilize this mechanism of action.

#### 4.2.1 Photoactivatable PROTACs

Photocaged and photoswitchable PROTACs offer high precision, enabling the “remote control” of therapeutic activity (e.g., CAR-T modulation) via light (Sharma et al., 2025). However, the “photon-penetration barrier” in deep tissues remains a significant translational hurdle, shifting current research toward near-infrared (NIR) and internal bioluminescence triggers.

#### 4.2.2 Enzyme- and microenvironment-activatable PROTACs

These “prodrug” degraders exploit the metabolic signatures of the tumor microenvironment (e.g., overexpressed O-GlcNAcase, nitroreductases, or GSH) for *in situ* activation (Ouyang et al., 2025; Zhu et al., 2025). While these systems vastly expand the therapeutic window, they are vulnerable to inter-patient enzymatic heterogeneity, suggesting that multi-signal “AND-gate” logic—requiring multiple pathological cues for activation—is the necessary next step for absolute selectivity (Dutta et al., 2025).

### 4.3 Delivery-enhanced platforms

#### 4.3.1 Antibody-PROTAC conjugates (APCs)

APCs harmonize the exquisite specificity of monoclonal antibodies with the catalytic power of TPD. By facilitating antigen-mediated endocytosis, APCs enable the delivery of potent degraders (e.g., Bromodomain-containing protein 4 (BRD4) or Bromodomain and Extra-Terminal domain family (BET) degraders) specifically to malignant cells, lowering the systemic effective dose and reducing collateral damage to healthy tissues (Maneiro et al., 2020; Zhao F. et al., 2025). Challenges include linker instability, incomplete tumor penetration, and the complexity of manufacturing such conjugates.

#### 4.3.2 Nano-PROTACs

Nanotechnology serves as a physicochemical buffer for PROTACs. Beyond improving solubility, advanced nano-platforms (e.g., Nano-CLIPTAC) achieve *in situ* assembly of the degrader within the cytosol. This not only circumvents the “Hook Effect” by controlling the local stoichiometry of the binary precursors but also enables the co-delivery of synergistic agents (Pan et al., 2025; Xie et al., 2025). Despite their promise, the translational path for Nano-PROTACs is complicated by manufacturing complexity (CMC hurdles) and the long-term metabolic fate of the synthetic nanocarriers. To help readers better understand the innovations in the development process of PROTAC technology, we have summarized this part of the content and presented it in the form of Table 1.

**TABLE 1:** Summary of PROTAC technical strategies.

Technology category	Subtype/Strategy	Key mechanism	Major advantages	Key challenges	Potential application context	References
Degrader architecture innovations	Multi-Target PROTACs	Single molecule degrades two distinct POIs, or a modular platform combines multiple ligands for complex degradation logic	• Simultaneously disrupts multiple disease-driving pathways • Potent synergy and potential to overcome redundancy-driven resistance • Accelerated discovery via modular design	• Molecular design is complex; Thermodynamic imbalance of binding affinities • Increased risk of on-target toxicity in healthy tissues due to broader pathway inhibition	• Therapy: Complex diseases like castration-resistant prostate cancer (targeting AR/BRD4) or endocrine-resistant breast cancer	Xin et al. (2024), Zhang et al. (2025)
	Multivalent PROTACs	Multiple ligand copies on a single scaffold (e.g., DNA tetrahedron) enhance binding avidity through cooperative effects	• Enhances degradation efficiency for oligomeric targets or weak-affinity ligands • Improves tumor targeting	• Increased risk of irreversible sequestration • Complex synthesis and purification	• Therapy: Targeting oligomeric proteins or proteins with weak individual ligands	Imaide et al. (2021), Li et al. (2025)
	Multi-E3 Ligase Recruitment	Simultaneous recruitment of two or more E3 ligases (e.g., CRBN and DCAF16) using monovalent or modular platforms	• Maintains degradation activity when one E3 pathway is compromised (redundancy). • Overcomes E3 ligase-mediated resistance	• Increased molecular complexity • Potential off-tissue toxicity	• Therapy: Tumors with heterogeneous E3 ligase expression; overcoming acquired resistance	Bond et al. (2024), Wang et al. (2025)
	Peptide-Based PROTACs	Utilizes peptide fragments to bind POIs, often targeting protein-protein interaction (PPI) interfaces	• Capable of targeting “undruggable” PPI interfaces and transcription factors (e.g., BRCA2, STAT3) • High binding specificity	• Compromised cytosolic delivery efficiency • Susceptible to proteolytic degradation	• Therapy: Targeting nuclear PPIs and transcription factors • Research: Protein function studies	Wang et al. (2024b), Ye et al. (2025)
	Nucleic Acid-Based PROTACs	Employs oligonucleotides (DNA, RNA, aptamers) for sequence-specific recognition of POIs	• High programmability and rapid retargeting • Suitable for “pocket-less” targets like transcription factors and RNA-binding proteins	• Very poor cellular uptake (requires delivery vehicle) • Nuclease susceptibility (requires chemical modification) • Challenging and expensive synthesis	• Therapy: Targeting RNA-binding proteins and transcription factors; personalized medicine	Hu and Crews (2022), Tsujimura et al. (2023), Xu et al. (2023), Hou et al. (2025)
	mRNA-Encoded PROTACs	*In vivo* synthesis of PROTAC (protein or peptide-based) via delivery of encoding mRNA.	• High programmability: target switching by mRNA sequence design • Bypasses cell permeability and “hook effect” limitations of small molecules • Enables sustained intracellular production	• mRNA requires chemical modifications and LNP delivery for stability and efficiency • Immunogenicity and long-term metabolic fate of LNPs • Transient expression profile	• Therapy/Research: Rapid prototyping for “undruggable” targets; potential for personalized neoantigen-targeted degradation	Yang et al. (2022a), Xue et al. (2024)
Conditional activation strategies	Photoactivatable PROTACs	Activity controlled by light-induced cleavage (photocaging) or isomerization (photoswitching) of integrated photoresponsive groups	• Exceptional spatiotemporal precision • Enables “on-demand” activation and reversible control • Minimizes systemic off-target exposure	• Limited tissue penetration of UV light • Potential biocompatibility issues with some photochromic moieties • Requires external device for activation	• Therapy: Potential for localized treatment (e.g., skin, accessible tumors) • Research: High-precision study of protein function *in vitro* and *in vivo*	Pfaff et al. (2019), Xue et al. (2019), Wang et al. (2023b), Sharma et al. (2025)
​	Enzyme-Activatable PROTACs	Activated selectively by disease microenvironment -overexpressed enzymes (e.g., NTR, cathepsin B, OGA) via prodrug cleavage or deglycosylation	• Endogenous trigger, no external device needed • Lesion-specific activation enhances therapeutic window • Reduces systemic toxicity	• Sensitivity to inter-patient enzymatic heterogeneity • Risk of off-target activation in normal tissues with basal enzyme levels	• Therapy: Precision oncology targeting hypoxic (NTR-high) or protease-rich tumors	Yang et al. (2023b), Ouyang et al. (2025), Zhu et al. (2025)
	Multi-Signal Responsive PROTACs	Requires simultaneous detection of multiple disease-associated cues for activation (AND-gate logic)	• Further improves specificity. • Substantially reduces off-target activation risk	• Increased molecular design complexity • Requires careful validation of each signal condition	• Therapy: Tumors with multiple distinct microenvironment features (e.g., low pH + high cathepsin B expression)	Liu et al. (2021), Yang et al. (2023a), Wang et al. (2024a), Dutta et al. (2025)
Delivery-enhanced platforms	Antibody-PROTAC Conjugates	Tumor-specific antibody delivers PROTAC payload intracellularly via receptor-mediated endocytosis and linker cleavage	• High tumor selectivity reduces on-target, off-tumor toxicity • Particularly suited for solid tumors with defined surface antigens	• Large molecular size may limit tumor penetration • Complex and costly manufacturing process • Linker stability and release kinetics require optimization	• Therapy: Solid tumors with well-defined surface markers (e.g., HER2+ breast cancer, CLL1+ leukemias)	Maneiro et al. (2020), Pillow et al. (2020), Zhao et al. (2025a)
	Nano-PROTACs	Nanocarriers (polymers, lipids) encapsulate or conjugate PROTACs for enhanced delivery and/or stimuli-responsive release	• Improves solubility, PK/PD properties, and tumor accumulation • Enables co-delivery for combination therapy (chemo/immuno) • Can be engineered for microenvironment-responsive release	• Manufacturing scalability and reproducibility are challenging • Potential carrier-related toxicity and immunogenicity • Heterogeneous distribution within tumors	• Therapy: Overcoming PK limitations of potent PROTACs; enabling combination regimens; targeting poorly vascularized tumors	Yang et al. (2024), Pan et al. (2025), Park et al. (2025), Xie et al. (2025)

## 5 The clinical translation of PROTAC technology

Since the inaugural clinical entry of ARV-110 in 2019, the PROTAC landscape has rapidly matured from mechanistic curiosity to late-stage therapeutic validation. The anticipated regulatory approval of ARV-471 (Vepdegestrant)—which submitted its New Drug Application (NDA) in mid-2025 following robust Phase 3 outcomes—represents a significant milestone for the field. However, regulatory approval remains subject to final review, and post-marketing safety surveillance will be critical. This milestone not only validates the systemic safety of chronic protein ablation but also establishes a regulatory precedent for “ bRo5” small molecules.

However, a critical appraisal of the current clinical pipeline reveals a stratified progress heavily favoring oncology. This “oncology-first” trajectory is driven by the urgent clinical need to overcome acquired resistance, such as the BTK C481S mutation in hematological malignancies or AR/ER ligand-binding domain (LBD) mutations in solid tumors. Yet, this progress is precariously anchored by an overwhelming CRBN dominance (>85% of clinical candidates), which poses a long-term risk of convergent resistance through E3-ligase genomic loss or transcriptional silencing.

Beyond the oncology stronghold, the transition into chronic, non-lethal indications like inflammatory diseases (e.g., KT-474/SAR444651 targeting IRAK4) and neurodegeneration (e.g., ARV-102 targeting LRRK2) marks a significant expansion of the TPD therapeutic ceiling. For these indications, the clinical bar for safety is substantially higher; the prolonged PD effect of PROTACs, while beneficial for efficacy, demands rigorous evaluation of irreversible immunosuppression or off-target neurotoxicity. Furthermore, optimizing oral bioavailability and tissue-specific exposure remains the primary pharmacokinetic hurdle for large-scale clinical adoption. The next wave of clinical translation should therefore pivot from merely achieving “target degradation” to mastering precision degradation—ensuring that the catalytic power of PROTACs is strictly confined to pathological tissues. Table 2 introduces representative PROTAC drugs currently in clinical trials.

**TABLE 2:** Summary of representative PROTAC drugs in clinical trials^a^.

Disease category	Drug name/Code	Sponsor(s)	Target	Indication(s)	E3 ligase	Highest global development phase
Oncology	Vepdegestrant (ARV-471)	Arvinas/Pfizer	ER	ER+/HER2- breast cancer	CRBN	NDA Under Review
	BGB-16673	BeiGene	BTK	CLL, B-cell Lymphomas	CRBN	Phase III
	BMS-986365 (Gridegalutamide)	Bristol-myers squibb	AR	Metastatic castration-resistant prostate cancer	CRBN	Phase III
	NX-5948 (Bexobrutinib)	Nurix	BTK	B-cell malignancies	CRBN	Phase I/II (pivotal ready)
	NX-2127 (Zelebrudomide)	Nurix	BTK	B-cell Lymphomas, leukemia	CRBN	Phase II
	ARV-110	Arvinas	AR	Prostate cancer	CRBN	Phase II
	ARV-766	Arvinas/Novartis	AR	Prostate cancer	VHL	Phase I/II
	CFT8919	C4 therapeutics/Betta pharma	EGFR L858 R	Non-small cell lung cancer	CRBN	Phase I/II
	MRT-2359	Monte rosa	GSPT1 (indirect MYC inhibition)	Solid tumors (e.g., Prostate cancer)	CRBN	Phase I/II
	RNK05047	Ranok therapeutics	BRD4	Diffuse large B-cell lymphoma	HSP90 chaperone complex	Phase I/II
	ARV-806	Arvinas	KRAS G12D	Pancreatic, colorectal, lung cancers	CRBN	Phase I
	ARV-393	Arvinas	BCL6	Non-hodgkin lymphoma	CRBN	Phase I
	GLR2037	Gan and lee pharmaceuticals	AR	Advanced prostate cancer	CRBN	Phase I
	HJ-004–02	Hejing medicine	EGFR (mutant)	Non-small cell lung cancer	CRBN	Phase I
	HRS-5041	Hengrui medicine	AR	Prostate cancer	CRBN	Phase I
	HSK38008	Haisco pharmaceutical	AR/AR-V7	Prostate cancer	CRBN	Phase I
	AC-0176	Accutar biotechnology	AR	Prostate cancer	CRBN	Phase I
	RO7656594	Roche/Arvinas	AR	Prostate cancer	CRBN	Phase I
	CC-94676	Bristol-myers squibb	AR	Prostate cancer	CRBN	Phase I
	HP-518	Hinova pharmaceuticals	AR	Prostate cancer	CRBN	Phase I
	PRT3789	Bristol-myers squibb	SMARCA2	SMARCA4-mutant NSCLC	VHL	Phase I
	DT2216	Dialectic therapeutics	BCL-XL	T-cell Lymphoma, solid tumors	VHL	Phase I
Immune and inflammatory	KT-621	Kymera therapeutics	STAT6	Atopic dermatitis, asthma	CRBN	Phase IIb
​	KT-474 (SAR444656)	Kymera/Sanofi	IRAK4	Atopic dermatitis, hidradenitis suppurativa	CRBN	Phase II
	MRT-6160	Monte rosa/Novartis	VAV1	Autoimmune diseases	CRBN	Phase II
	HPB-143	Glubio	IRAK4	Inflammatory diseases	CRBN	Phase I
	KT-579	Kymera therapeutics	IRF5	Lupus, rheumatoid arthritis	CRBN	Phase I (imminent)
	KT-485 (SAR447971)	Kymera/Sanofi	IRAK4	Immuno-inflammatory diseases	CRBN	Phase I (imminent)
	MRT-8102	Monte rosa	NEK7	NLRP3 Inflammasome-associated diseases	CRBN	Phase I
Neurodegenerative	ARV-102	Arvinas	LRRK2	Parkinson’s disease, progressive supranuclear palsy	CRBN	Phase I
	ARV-027	Arvinas	polyQ-AR	Spinal and bulbar muscular atrophy	CRBN	Phase I

^a^ Data were sourced from ClinicalTrials.gov (https://clinicaltrials.gov/) and the Chinese Clinical Trial Registry (http://www.chinadrugtrials.org.cn); AR (Androgen Receptor), AR-V7 (Androgen Receptor Splice Variant 7), BCL6 (B-cell Lymphoma 6 protein), BTK (Bruton’s Tyrosine Kinase), CLL (Chronic Lymphocytic Leukemia), EGFR (Epidermal Growth Factor Receptor), ER (Estrogen Receptor), HER2 (Human Epidermal Growth Factor Receptor 2), HPK1 (Hematopoietic Progenitor Kinase 1), HSP90 (Heat Shock Protein 90), IRAK4 (Interleukin-1 Receptor-Associated Kinase 4), IRF5 (Interferon Regulatory Factor 5), KRAS G12D (Kirsten Rat Sarcoma Viral Oncogene Homolog with G12D mutation), LRRK2 (Leucine-Rich Repeat Kinase 2), NEK7 (NIMA-Related Kinase 7), NLRP3 (NLR Family Pyrin Domain Containing 3), polyQ-AR (Polyglutamine-Expanded Androgen Receptor), SMARCA2 (SWI/SNF-Related Matrix-Associated Actin-Dependent Regulator of Chromatin Subfamily A Member 2), SMARCA4 (SWI/SNF-Related Matrix-Associated Actin-Dependent Regulator of Chromatin Subfamily A Member 4), STAT6 (Signal Transducer and Activator of Transcription 6), and VAV1 (Vav Guanine Nucleotide Exchange Factor 1).

## 6 Summary and outlook

Since its inception in 2001, PROTAC technology has undergone a significant trajectory, transitioning from rudimentary peptide-based probes to an advanced clinical modality. By subverting the traditional occupancy-driven paradigm with an event-driven catalytic mechanism, PROTACs have demonstrated the potential to eliminate historically “undruggable” proteins and circumvent certain acquired resistance mechanisms. While the anticipated regulatory approval of candidates like ARV471 (Vepdegestrant) provides compelling evidence for the translational viability of this platform, the transition from “functional degradation” to “clinical superiority” remains a complex endeavor.

Current research frontiers reflect a transition toward enhanced structural intelligence and spatiotemporal precision. Conditionally activatable PROTACs are beginning to resolve the inherent tension between systemic exposure and on-target/off-tumor toxicity, providing a molecular “gatekeeper” for high-potency degradation. Concurrently, the integration of advanced delivery platforms—most notably APCs and mRNA-encoded degraders—is systematically addressing the “bRo5” pharmacokinetic penalties associated with large-molecule degraders. These innovations are not merely technical iterations but represent a fundamental shift toward network pharmacology, where multiple pathogenic nodes can be dismantled with modular, programmed efficiency. Nevertheless, the clinical translation of these delivery platforms faces its own challenges, including manufacturing complexity and long-term safety.

Looking ahead, the evolution of PROTAC technology must confront several unresolved bottlenecks. First, the current “E3 ligase monopoly” (primarily CRBN and VHL) poses a significant risk of convergent resistance; the discovery of tissue-specific or tumor-selective E3 ligands is therefore paramount. Second, the predictive modeling of ternary complex dynamics requires a deeper synergy between artificial intelligence (e.g., Deep Learning) and high-resolution structural biology. Third, the long-term metabolic fate and safety profiles of novel delivery vehicles require rigorous clinical scrutiny.

Ultimately, PROTAC technology is poised to evolve from a specialized drug-discovery tool into a programmable, customizable targeted degradation platform. As the field expands beyond oncology into neurodegeneration, autoimmunity, and infectious diseases, the focus will shift from “generalized protein knockdown” toward precision-guided personalized medicine. By harmonizing molecular engineering with a sophisticated understanding of cellular proteostasis, PROTACs will continue to expand the therapeutic scope of modern medicine.

## Glossary

APC: Antibody-PROTAC Conjugates
AR: Androgen Receptor
BBB: Blood–Brain Barrier
BET: Bromodomain and Extra-Terminal domain
BRCA2: Breast Cancer Susceptibility Gene 2
BRD4: Bromodomain-containing Protein 4
BTK: Bruton’s Tyrosine Kinase
bRo5: Beyond Rule of 5
CAR-T: Chimeric Antigen Receptor T-cell
CRBN: Cereblon
ER: Estrogen Receptor
IRAK4: Interleukin-1 Receptor-Associated Kinase 4
irAEs: immune-related adverse events
LBD: ligand-binding domain
LRRK2: Leucine-Rich Repeat Kinase 2
MAPK: Mitogen-Activated Protein Kinase
NF-κB: Nuclear Factor kappa-light-chain-enhancer of activated B cells
PD: pharmacodynamic
PD-1: Programmed Death-1
PD-L1: Programmed Death-Ligand 1
PK: pharmacokinetic
POI: Protein of Interest
PPI: Protein-Protein Interaction
PROTAC: Proteolysis-Targeting Chimera
PSMA: Prostate-Specific Membrane Antigen
PCSK9: Proprotein Convertase Subtilisin/Kexin type 9
SMAD: Suppressor of Mothers Against Decapentaplegic
TPSA: topological polar surface areas
TPD: Targeted Protein Degradation
UPS: Ubiquitin-Proteasome System
VHL: Von Hippel-Lindau
